# Mobile CRISPR/Cas-Mediated Bacteriophage Resistance in *Lactococcus lactis*

**DOI:** 10.1371/journal.pone.0051663

**Published:** 2012-12-11

**Authors:** Anne M. Millen, Philippe Horvath, Patrick Boyaval, Dennis A. Romero

**Affiliations:** 1 DuPont Nutrition and Health, Madison, Wisconsin, United States of America; 2 DuPont Nutrition and Health, Dangé-Saint-Romain, France; Belgian Nuclear Research Centre SCK/CEN, Belgium

## Abstract

*Lactococcus lactis* is a biotechnological workhorse for food fermentations and potentially therapeutic products and is therefore widely consumed by humans. It is predominantly used as a starter microbe for fermented dairy products, and specialized strains have adapted from a plant environment through reductive evolution and horizontal gene transfer as evidenced by the association of adventitious traits with mobile elements. Specifically, *L. lactis* has armed itself with a myriad of plasmid-encoded bacteriophage defensive systems to protect against viral predation. This known arsenal had not included CRISPR/Cas (clustered regularly interspaced short palindromic repeats/CRISPR-associated proteins), which forms a remarkable microbial immunity system against invading DNA. Although CRISPR/Cas systems are common in the genomes of closely related lactic acid bacteria (LAB), none was identified within the eight published lactococcal genomes. Furthermore, a PCR-based search of the common LAB CRISPR/Cas systems (Types I and II) in 383 industrial *L. lactis* strains proved unsuccessful. Here we describe a novel, Type III, self-transmissible, plasmid-encoded, phage-interfering CRISPR/Cas discovered in *L. lactis*. The native CRISPR spacers confer resistance based on sequence identity to corresponding lactococcal phage. The interference is directed at phages problematic to the dairy industry, indicative of a responsive system. Moreover, targeting could be modified by engineering the spacer content. The 62.8-kb plasmid was shown to be conjugally transferrable to various strains. Its mobility should facilitate dissemination within microbial communities and provide a readily applicable system to naturally introduce CRISPR/Cas to industrially relevant strains for enhanced phage resistance and prevention against acquisition of undesirable genes.

## Introduction

*Lactococcus lactis* is a lactic acid bacterium (LAB) indispensable for the production of approximately 40 million metric tons of fermented dairy foods annually, which represents a hundreds of billions (USD) dollar global industry (Euromonitor Passport 2011 report). Beyond preserving a perishable food and providing a safe source of human nutrition, fermented dairy products are a vehicle for the consumption and dissemination of billions of lactococci into the human and environmental microbiome.

In nature, lactococci are believed to inhabit a plant environmental niche [1]. The chance contamination by variants able to grow in milk has advanced into the rigorous industrial selection and development of highly adapted strains that are essential for today’s processing demands. These specialized strains, referred to as starter cultures, possess unique metabolic properties responsible for the diversity of fermented dairy products. Today, a principal criterion for starter strain selection is the strain’s ability to resist virulent phage predation, a major cause of failed dairy fermentations whichresult in significant waste and economic loss. Effective lactococcal starter strains have naturally developed an extensive array of defensive mechanisms to combat phage infection [2]. Many are plasmid-encoded, and often multiple complementary mechanisms are combined on a single element and coupled with conjugative transfer functions. These genetic features have been exploited to protect uniquely valuable strains [2], [3]. Notably absent, however, were CRISPR/Cas systems [4].

CRISPRs (clustered regularly interspaced short palindromic repeats) are widely disseminated in bacteria and archaea [for recent reviews see 5, 6, 7, 8, 9]. They are composed of repeat sequences separated by unique intervening sequences (spacers) that are generally derived from viral and plasmid sequences. In many cases, a group of CRISPR-associated (*cas*) genes are found adjacent to the CRISPR array. The two components form a functional pair that confers immunity based on spacer sequence identity against foreign DNA, including bacteriophage and plasmids. No CRISPR or *cas* gene was identified within the current eight publicly available lactococcal genomes [10]. A PCR-based search in 383 industrial *L. lactis* strains from the DuPont collection did not reveal any Type I or Type II CRISPR/Cas system, which are common to LABs [4]. Here we describe a novel CRISPR/Cas system in *L. lactis*, discovered in the course of searching for novel phage resistance mechanisms.

## Methods

### Bacteria, Bacteriophages, Plasmids, and Culturing Conditions

Bacterial strains, phages, and plasmids are listed in Table 1. All *L. lactis* were grown at 30°C in M17 broth (Becton, Dickenson and Co., MD, USA) supplemented with 0.5% lactose (Lac) or glucose (Glu). *Escherichia coli* was propagated aerobically in LB broth (Becton, Dickenson and Co., MD, USA) at 37°C. When required, antibiotics were added to the media as follows: streptomycin (Sm, 1000 µg/ml), spectinomycin (Sp, 300 µg/ml), and erythromycin (Em, 5 µg/ml). Plasmid curing was performed by sequential transfer at 37°C in M17Glu. Selection of cured isolates for loss of lactose fermenting ability was done by plating serial dilutions on BCP Lactose Indicator agar containing bromocresol purple and 1% lactose [11]. For Em curing, individual colonies were first isolated on M17Glu then replica plated onto media with and without Em. Preparation of bacteriophage lysates was performed as described by Terzaghi and Sandine [12]. Lysates were passed through a 0.45 µm filter and stored at 4°C. Plaque assays were performed as described by Terzaghi and Sandine [12] on MRS medium with the exception of phage 949 assays which were performed on MRS +0.5% glycine.

**Table 1:** List of bacterial strains, plasmids, and bacteriophages.

Strains, Plasmids, Phages	Relevant Characteristics	Description/Reference
***L. lactis***		
DGCC7167	Lac+	DuPont collection, industrial starter
IL1403	Lac−, plasmid-free	Conjugation host, Accession AE005176 [38]
1403S	Lac−, SmR, plasmid-free,	Spontaneous SmR derivative of IL1403
LM2302	Lac−, SmR EmR, plasmid-free	Conjugation recipient host [39]
LM2345	Lac−, SpR RfR, plasmid-free	Conjugation recipient host [40]
DGCC7192	Lac+	DuPont collection, industrial starter, recipient host
K	Lac−, SmR EmR, at least 4 plasmids	1403S transconjugant (DGCC7167 conjugation donor)
**Plasmids**		
pGhost9	EmR, TS ori (pGh9)	Temperature sensitive vector [29]
pGhost9::IS*S1*	EmR, TS ori ISS*1* (pGh9::IS*S1*)	Insertion sequence IS*S1* variant of pGhost9 [29]
pGK12	EmR CmR	Broad host range vector [41]
pCR4Blunt-TOPO	KmR ApR cloning vector	Life Technologies Corp, USA
pKLM	Tra+ CRISPR/Cas EmR	Fusion of DGCC7167 native plasmid with pGh9::IS*S1*
pLN	Tra+ CRISPR/Cas	Lac-cured CRISPR plasmid from DGCC7167
pF8E	Tra+ CRISPR/Cas EmR	pLN::pGh9::IS*S1* with IS*S1* inserted into s9 of pLN
pG6E	Tra+ CRISPR/Cas EmR	pLN::pGh9::IS*S1* Δs2-s9
pG6	Tra+ PhageR	Resolved pLN::pGh9::IS*S1* Δs2-s9 with loss of pGh9::IS*S1*
pTOPOS4	repeat::s4::repeat (RS4R)	RS4R construct cloned into pCR4Blunt-TOPO (Invitrogen, CA, USA)
pRS4R	EmR	pTOPOS4 cloned into pGhost9
pG6::pRS4R	EmR, cointegrate of pG6 and pRS4R	Fusion of pRS4R into CRISPR of pG6
**Bacteriophages**		
p2	Host LM2301/LM2302/LM2345	Type 936, Accession GQ979703
bIL67	Host IL1403/1403S	Type c2, Accession L33769 [42]
bIL170	Host IL1403/1403S	Type 936, Accession AF009630 [43]
949	Host IL1403/1403S	Accession HM029250 [44]
P008	Host IL1403/1403S	Type 936, Accession DQ054536 [45]
P335	Host IL1403/1403S	Accession DQ838728 [46]
M5952	Host DGCC7192	DuPont collection, 4268-like (4268 Accession AF489521) [23]

(+) = positive phenotype/(−) = negative phenotype/(R) = resistant.

Lac = lactose fermentation/Sm = streptomycin/Em = erythromycin.

Cm = chloramphenicol/Sp = spectinomycin/Rf = rifampicin.

Km = kanamycin (*E. coli* only)/Ap = ampicillin (*E. coli* only).

TS ori = temperature sensitive origin of replication.

Tra = conjugative transfer.

Repeat = lactococcal CRISPR repeat sequence.

### Electroporation and Conjugation

Electroporation of pGh9::IS*S1* into *L. lactis* was performed according to the method of Holo and Nes [13]. Solid surface conjugal mating was performed as described by McKay et al. [14] except that M17 medium containing 0.5% glucose or 0.5% glucose and lactose was substituted for milk agar plates. Transconjugants were selected on M17 or BCP Lactose indicator agar media supplemented with the respective carbohydrate, antibiotics, or phage.

### DNA Manipulation

Lactococcal plasmid DNA was prepared by the method of Anderson and McKay [15]. Preparative amounts of pKLM DNA for sequencing were purified through cesium chloride-ethidium bromide density gradient centrifugation for at least 20 h at 15°C and 57,000 *g* using a Beckman L8-60M ultracentrifuge and NVT65 rotor. For *E. coli*, DNA was purified using a QIAprep Spin Miniprep Kit (Qiagen, Hilden Germany). Primers used in this study are listed in Table S1 and were designed from pKLM or phage 4268 sequences. Cell pellets resuspended in sterile water were used as template for amplification. Purified lysate (10^9^ plaque forming units/ml) was used as template for phage amplification. PCR was performed with GoTaq DNA polymerase (Promega, WI, USA). Reactions were set up per manufacturer’s instructions. Thermal cycler conditions were as follows: initial denaturation for 5 min at 94°C followed by 30 cycles of denaturation at 94°C for 30 s, annealing at 51°C for 30 s, and extension at 72°C (time dependent based on amplicon size, 30–45 seconds per kb), then final extension at 72°C for 5 min. PCR products were purified using the QIAquick PCR Purification Kit (Qiagen, Hilden Germany).

### DNA Sequencing and *in silico* Analysis

The plasmid sequence for pKLM was obtained from the Roy J. Carver Biotechnology Center (University of Illinois Urbana-Champaign, IL) by utilizing FLX-Titanium 454 sequencing [16]. A total of 10,233 reads were generated with an average coverage of 67X. A *de novo* assembly was generated using NGen (DNAstar, Madison, WI) software and was subsequently inspected for quality using SeqMan Pro (DNAstar, Madison, WI). Gaps in sequence were closed by PCR, and standard dye terminator sequencing was performed by Northwoods DNA, Inc (Solway, MN) using amplification primers. Annotation was performed using BLASTn and BLASTx [17]. Plasmid map and sequence comparison were created using BLAST Ring Image Generator (BRIG) [18]. The pKLM CRISPR/Cas sequence is available under GenBank accession number JX524189.

### Natural CRISPR Adaptation

Phage challenges were performed essentially by standard plaque assay [12] on phage sensitive CRISPR/Cas-containing strains. Bacteriophage insensitive mutant (BIM) colonies were tested for spacer addition by PCR. BIM CRISPR arrays were amplified using primers CR-F2 and CR-R3B (Table S1). Spacer addition would be indicated by an increase in amplicon size. Plasmid stability assays were performed essentially as described by Garneau et al. [19]. pGK12 (EmR) was first electroporated into IL1403S and 1403S (pLN). Cultures were propagated at 37°C in M17Glu then plated after 12 transfers on M17 Glu ± Em.

### Insertional Mutagenesis

pGh9::IS*S1* was electroporated into IL1403S containing pLN. Transformants were used as donors in a conjugation with LM2345. Em resistant, phage p2 sensitive transconjugants were amplified with primers designed from the *cas* genes and CRISPR array (Table S1). Amplicons larger than the pKLM control were sequenced to determine the location of IS*S1* insertion.

### Spacer Engineering

A synthetic, single spacer CRISPR array was constructed via successive PCR reactions using Finnzymes Phusion High-Fidelity DNA Polymerase (Thermo Fisher Scientific, Vantaa, Finland). pG6 was amplified with primer set F1 and S4R and separately with primer set IS1194 and S4F (Table S1). Amplicons were cleaned up with the QIAquick PCR Purification Kit (Qiagen, Hilden Germany) then mixed 1∶1. PCR was performed on the amplicon mix using primer set F1 and IS1194. Thermal cycler conditions for each reaction were as follows: initial denaturation for 30 s at 98°C followed by 30 cycles of denaturation at 98°C for 10 s, annealing at 51°C for 30 s, and extension at 72°C (time dependent on amplicon size, 10–30 seconds per kb), then final extension at 72°C for 5 min. The resulting amplicon spanned the 3′ end of *cas1* through the truncated IS*1194* fragment with a repeat-s4-repeat CRISPR sandwiched in-between. The construct was first cloned into pCR4Blunt-TOPO (Invitrogen, CA, USA) and transformed into chemically competent One Shot TOP10 *E. coli* (Invitrogen, CA, USA). Plasmids were isolated from *E. coli* using the QIAprep Spin MiniPrep Kit (Qiagen, Hilden Germany). To enable selection and replication in a lactococcal host, the pCR4Blunt-TOPO containing the desired insert (pS4TOPO) was fused to the lactococcal vector pGhost9 (pGh9). pS4TOPO and pGh9 were each cut with SpeI (Promega, WI USA). The cut vectors were ligated using T4 DNA ligase (Invitrogen, CA, USA). All reactions were performed according to manufacturer’s instructions. The resulting fusion plasmid was designated pRS4R, and the integrity of the synthetic CRISPR was confirmed by DNA sequencing. pRS4R was electroporated into lactococcal host 1403S containing pG6. To ensure recombination with the CRISPR/Cas present on pG6, 1403S containing pG6 and pRS4R was used as a conjugal donor for EmR mobilization to plasmid-free recipient LM2345.

## Results

### Plasmid-encoded, Self-transmissible Phage Resistance

**Figure 1:**
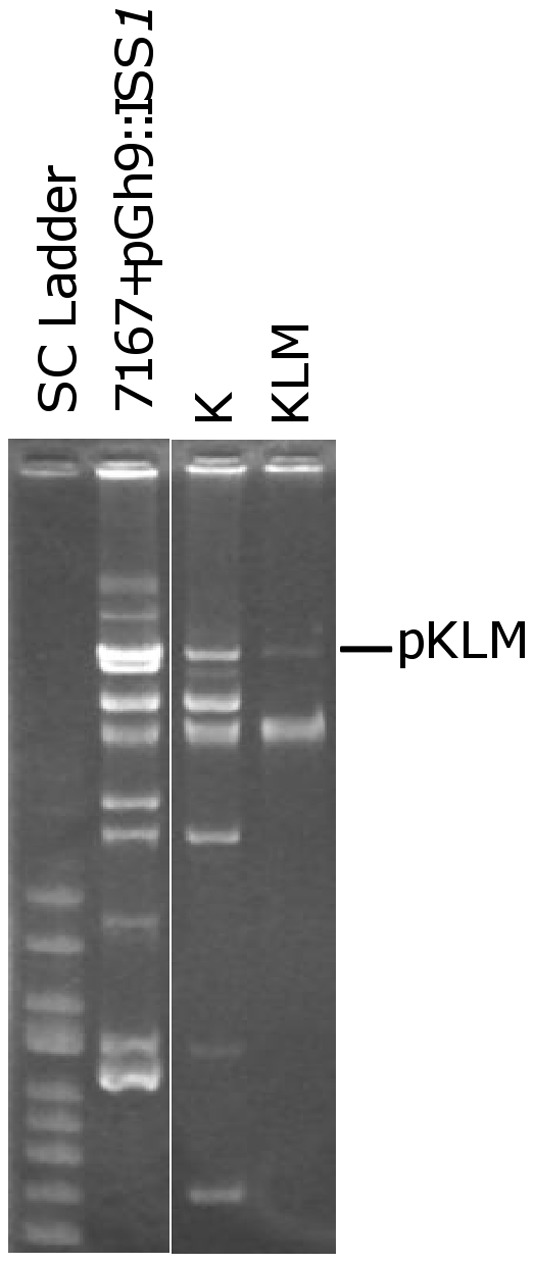
Plasmid profiles. Plasmid profile of donor *L. lactis* DGCC7167 + pGh9::IS*S1*, phage resistant transconjugants K (derived from recipient *L. lactis* 1403S) and KLM (derived from recipient LM2345).

*L. lactis* DGCC7167, which showed a high level of natural phage resistance, was selected as a potential donor of conjugative plasmid-encoded phage resistance. To circumvent generating spontaneous phage resistance mutants, we chose to first introduce pGh9::IS*S1* into the donor strain to facilitate co-integration with native self-transmissible elements. Erythromycin resistance (EmR), encoded by pGh9::IS*S1*, could then be used as selection for conjugal mobilization. EmR was transferred to plasmid-free *L. lactis* 1403S, and transconjugants were screened for resistance to *L. lactis* phages 949, bIL67, bIL170, and P335. The latter three are representative of the problematic phage types (types c2, 936, and P335, respectively) in the dairy industry. Following conjugal transfer at approximately 10^−7^ per exit recipient, one transconjugant (designated K) amongst those screened was resistant to all phages tested and harbored multiple plasmids (Figure 1). To determine if one of these plasmids encoded phage resistance, K was conjugally mated to another plasmid-free recipient, *L. lactis* LM2345. Transfer occurred at 4-log higher efficiency. A representative transconjugant (designated KLM) that was resistant to phage p2 was selected and found to contain a single plasmid of approximately 60 kb, corresponding to one of the plasmids found in donor K (Figure 1). This plasmid, designated pKLM, was transferred concomitantly with phage resistance in second round conjugal matings from donor KLM (10^−4^ per exit recipient). In addition, loss of pKLM returned a strain to phage sensitivity. These two lines of evidence provide proof that pKLM encodes the phage resistance phenotype. Based on these results, pKLM was purified and sequenced.

### Plasmid pKLM Harbors a Novel CRISPR/Cas System

**Figure 2:**
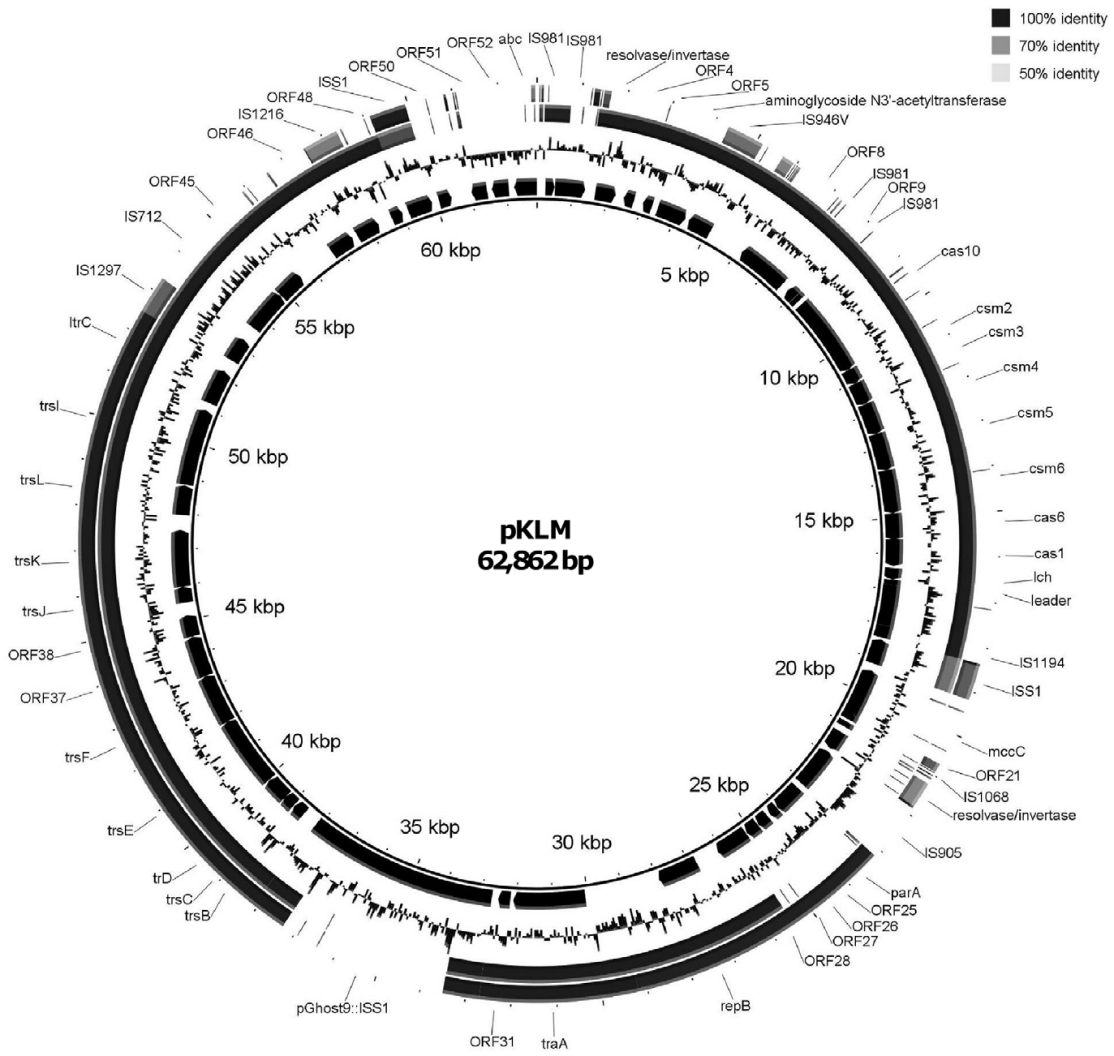
pKLM map and plasmid comparison. Rings from inside out: pKLM ORFs; pKLM GC content; nucleotide identity with pLN; nucleotide identity with pMRC01.

The pKLM sequence revealed a 62,862-bp plasmid encoding a novel CRISPR/Cas locus which spans a 9.6-kb segment (Figure 2). The CRISPR array is composed of 16 identical 36-nt repeats interspaced by 15 spacers ranging in size from 33 to 39 nt. The repeat (5′-AAATACAACCGCTCCTCGATAAAAGGGGACGAGAAC-3′) shows size and sequence similarity to those belonging to CRISPR/Cas Type III-A [7]. In particular, the 3′ terminal 16-nt of the lactococccal repeat matches perfectly with *Staphylococcus epidermidis* RP62a [20] and near perfectly (15 of 16-nt) to *Enterococcus italicus* DSM 15952 (accession number AEPV01000074) 3' termini. In *S. epidermidis*, it has been shown that the 3′ repeat terminus, specifically the sequence GGGACG, is critical for cleavage of CRISPR RNAs (crRNA) by Cas6 [21], [22]. Sequence analysis of the 15 spacers using BLASTn found 7 with partial identity to known lactococcal phages (Table 2). Four spacers (s5, s8, s10, s13) match phage 949, and three spacers (s2, s3, s4) match 936-type phages that include p2, bIL170, and P008. It is noteworthy that these spacers correspond to the resistance phenotypes against the respective phage types. Spacer s3 also shows a match to lytic phage 4268 [23] and prophage BK5-T [24].

**Table 2:** List and nucleotide sequence of pKLM/pLN CRISPR spacers.

Spacer	Length (nt)	Sequence	Phage Match	nt Match to Phage
s15	35	TGCATGTTTATAGCCCTGCCGGATTTTAAGCTGCG		
s14	38	TTTCCATTCCGTTTAACTGCTGCCAGAAAGATTTCATC		
s13	38	TGGTTGTTGTCATTAGCTGTATCGTGAATGACGATATA	949	36
s12	34	AACTTGGAATGGTAATTCATATAATTTTTTCATA		
s11	35	TGCTGGTTTTATTTGCTCAATTTTTGAATTGTCAA		
s10	39	TTTGTTGTAAAATATTTCATGTTTTGTTTTCTCTTTTCT	949	37
s9	38	AGAGAGTATTCAGTCATGAATGAAATGATTGCAATTTG		
s8	35	ACAACTGTTTTAACTCTATCCTGATATATAAACCC	949	26
s7	33	AACTTTTTAAGGATAAGACCAACAGACTCTGAC		
s6	37	TTTATTTGTGGCAACAAGTTCAGCAATAATAGGGTTT		
s5	37	GAACTTAGCAAGCTATTTTGTTTCTTTTCAAGAGCCA	949	32
s4	35	ATACGTTCTTTGAACCAAGCTTCAACTCCCTC_GGA*	p2	32
		ATACGTTCTTTGAACCAAGCTTCAACTCCCTC_GGA*	P008	32
		ATACGTTCTTTGAACCAAGCTTCAACTCCCTC_GGA *	bIL170	28
s3	35	TTCTGTTAATTTAACTCCCATTTGTTAGTTCTCCT	p2	32
		TTCTGTTAATTTAACTCCCATTTGTTAGTTCTCCT	P008	28
		TTCTGTTAATTTAACTCCCATTTGTTAGTTCTCCT	4268	26
s2	35	TTTTTAAAATGTTGCAAATGTTTAGCTACTTCAT	P008	33
s1	34	ATATGTCGGTTTGTCTTTTGGTCTAACGTATGCA		

nt = nucleotides.

Underlined nucleotides indicate mismatches against respective proto-spacer target.

* A single nucleotide gap denoted by an underscore space in sequence.

**Figure 3:**
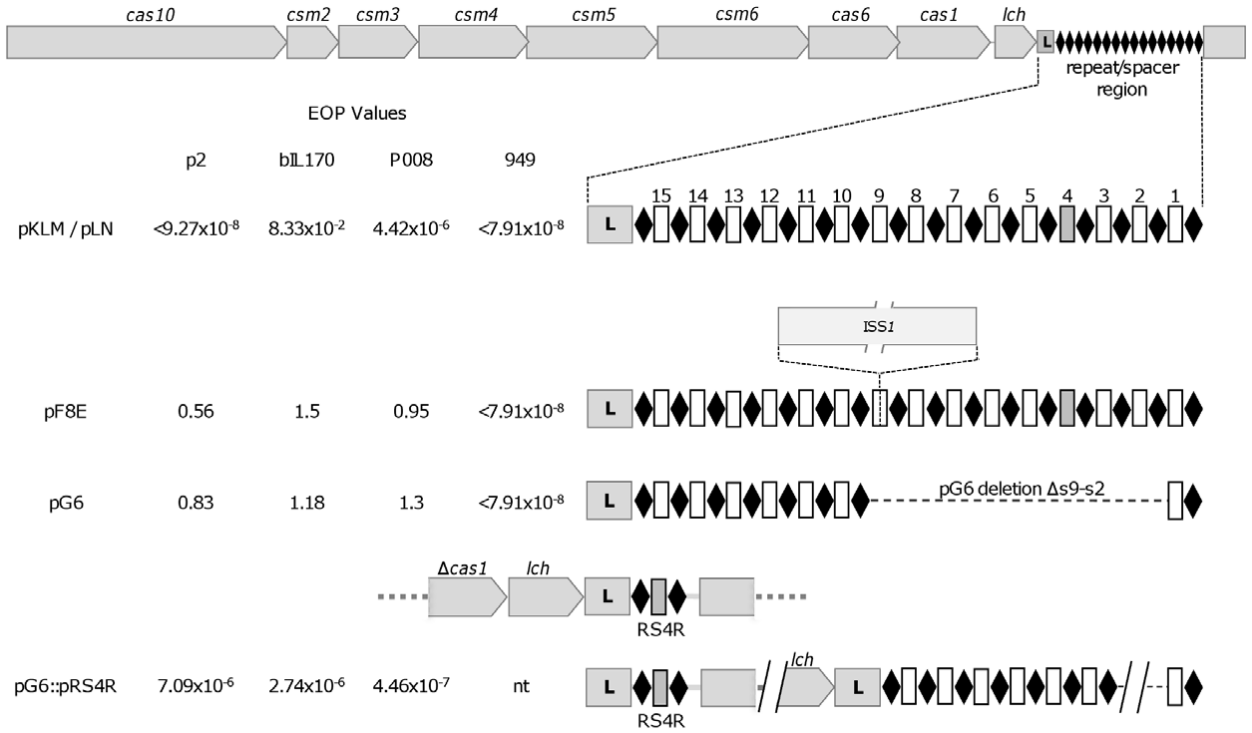
Expanded view of the CRISPR/Cas locus of pKLM/pLN, pF8E, pG6, and pG6::pRS4R. View of *cas* genes and CRISPR arrays correlated to phage resistance phenotype. pKLM/pLN contains the full spacer array which gives resistance to all 4 listed phages. pF8E contains an insertion within the array which disrupts spacers s1-s9 as well as resistance to phage encoded by those spacers. pG6 contains a deletion of spacers s2-s9, and resistance to phage encoded by those spacers is lost. pG6::pRS4R contains an engineered s4 which provides resistance to all 3 phages it has identity to.

Adjacent to the CRISPR array is a set of 9 colinear genes. Eight of the 9 encode proteins which are similar to Cas and Csm proteins of Type III-A systems [7] (Figure 3). These 8 genes include (in sequence on the locus) *cas10*, *csm2*, *csm3*, *csm4*, *csm5*, *csm6*, *cas6,* and *cas1*. Interestingly absent is a *cas2* homologue, which is an endoribonuclease believed to participate in spacer acquisition with *cas1* [25]. In its place and immediately ensuing *cas1*, is a 333-nt open reading frame (ORF) with 55% amino acid similarity to the RelE family toxin component of toxin-antitoxin systems involved in plasmid stabilization [26]. Sequence analysis identifies this ORF, which we have designated *lch*, as having a conserved domain belonging to the pfam05016/TIGR02385 family of proteins that are addiction module toxins involved in plasmid stabilization [27].

A 150-nt putative leader sequence is found between *lch* and the first CRISPR repeat. Some CRISPR arrays are delineated by a terminal degenerate repeat, which is not the case here. A 35-nt segment without sequence similarity follows the distal repeat. This segment abuts a vestigial IS*1194* transposase, which itself contains an insertion of a defective IS*S1* element. This suggests that the CRISPR array has been interrupted by prior recombination events.

### Other Genetic Features of pKLM

The remainder of pKLM corresponds primarily to lactococcal plasmid pMRC01 conjugative (*orf5-20* and *ori*T) and replication (*orf62*, *repB*) functions [28], and it contains ten transposase genes (five IS*S1* elements, single copies of IS*905*, IS*981*, IS*1216*, IS*1217*, and IS*712A*, and several IS remnants). pGh9::IS*S1* transposed into the counterpart of pMRC01 hypothetical gene *orf7* accounts for two of the IS*S1* copies (Figure 2).

Overall, pKLM has a G+C content of 31.8% after subtracting pGh9::IS*S1*. This compares to the G+C content of lactococcal genomes which averages 35.5%, and to 32.2% for *S. epidermidis* RP62a. The pKLM CRISPR/Cas locus by itself has a 34.7% G+C versus 29.0% and 37.3% for the loci in *S. epidermidis* RP62a and *E. italicus* DSM 15952, respectively.

### Food-Grade CRISPR/Cas Plasmid pLN

A food-grade, non-antibiotic marked plasmid containing CRISPR/Cas from donor DGCC7167 was isolated followinga conjugation with plasmid-free strain LM2302 byselecting for the mobilization of lactose fermenting ability (Lac). A representative phage resistant, Lac-positive transconjugant was selected. The Lac phenotype was then cured to prevent any potential incompatibility with native Lac plasmids when conjugated into industrial starter strains. The Lac-cured strain contained a single plasmid of about 60 kb, designated pLN. pLN was conjugative with concomitant transfer of phage resistance. A draft sequence of pLN was generated. About 78% of the pLN draft sequence was conserved with pKLM (Figure 2). The presence of CRISPR/Cas identical to the KLM CRISPR/Cas was confirmed.

### Cas Proteins are Necessary for Phage Resistance

Insertional mutagenesis of pLN with IS*S1* [29] was used to investigate *cas* gene involvement in phage resistance. EmR was conjugally mobilized at 7×10^−5^ per exit recipient. At least one example of IS*S1* insertion was identified for seven of the *cas* genes and *lch* in pLN. In each case, the respective transconjugant was sensitive to phage p2. No insert was found in *cas6* or the leader region in the 36 phage p2-sensitive isolates examined. These results confirm the involvement of the *cas* gene cluster in phage resistance. The possibility of polar transcriptional effects was not ruled out, which would be consistent with the observations that insertionally inactivated *cas1* and *lch*, which are not believed to be involved in interference, lead to phage sensitivity.

### Phage Resistance is Directed by CRISPR Spacers

Spacer sequence is directly correlated to CRISPR/Cas-mediated immunity to phage [30]. Two phage p2-sensitive variants were characterized in the insertional mutagenesis experiments in which IS*S1* had not inserted into the *cas* gene cluster. In the first variant, pGh9::IS*S1* insertion was not conclusively mapped, however, its single plasmid (designated pG6E) was found to have precisely deleted spacers s9 through s2 (Figure 3). In the second plasmid (pF8E), IS*S1* had inserted into the CRISPR array, specifically into spacer s9, which displaced spacers s1 through s8 relative to the *cas* genes and leader. Consequently, IS*S1* insertion would disrupt transcription of the displaced spacers in the array (Figure 3). In both cases, p2 sensitivity can be correlated to the deletion or displacement of spacers s3 and s4, which have partial identity to phage p2.

Spacers s3 and s4 also have partial sequence identity to phages P008 and bIL170 (Table 2). When plasmid pG6E or pF8E was conjugally transferred to the plasmid-free host 1403S, transconjugants remained sensitive to phages P008 and bIL170. In contrast, these 1403S transconjugants were resistant to phage 949, which is targeted by spacers s5, s8, s10, and s13. Spacers s10 and s13 are retained in pG6E and remain in proper transcriptional context in pF8E. Taken together, these results further support the involvement of spacers and a level sequence identity that is yet to be determined in phage interference.

As noted, spacer s3 also shows partial identity to lactococcal phage 4268 (Table 2). DuPont collection phage M5952 had previously been sequenced and was found to be closely related to phage 4268 (data not shown), sharing identical sequence across the proto-spacer and flanking regions. CRISPR/Cas interference against M5952 was tested after conjugal transfer of pKLM or pLN into the industrial starter strain *L. lactis* DGCC7192. Conjugal transfer of EmR or phage resistance was observed, and each plasmid conferred resistance to phage M5952. This suggests that spacer s3 could direct the interference phenotype. In a plaque assay, distinct plaques were observed at the lowest phage dilutions tested, which indicates that some phages had escaped the CRISPR/Cas immune system.

### Analysis of Phage Escape Mutants

In *Streptococcus thermophilus*, phages that escape CRISPR/Cas immunity are found to have a mutation within the corresponding phage genome sequence (proto-spacer) or PAM (proto-spacer adjacent motif), which is also important for the interference phenotype [30], [31], [32]. In the course of testing DGCC7192(pKLM), escape phages derived from M5952 were observed at approximately 10^−6^. Seventy-three escape phages were examined by sequencing a 438-bp amplicon spanning the s3 proto-spacer. Each escape phage was found to have a single nucleotide mutation, mapping to 5 positions within the 22-nt identity segment of spacer s3 (Table 3). The isolation and characterization of escape phages provide further proof of spacer-directed interference.

**Table 3:** Escape phage analysis.

	Sequence	Number of Isolates
**s3**	TTCTGTTAATTTAACTCCCATTTGTTAGTTCTCCT	
**A**	CTGTTAATTTAACT**T**CCATTTG	5
**B**	CT**A**TTAATTTAACTCCCATTTG	49
**C**	CTGTTAATTTAACTCCCAT**G**TG	1
**D**	CT**T**TTAATTTAACTCCCATTTG	8
**E**	CTGTTAATTTAAC**A**CCCATTTG	3
**F**	CTGTTAATTTAACT**G**CCATTTG	3
**G**	CTGTTAATTT**G**ACTCCCATTTG	1
**H**	CTGTTAATTTAAC**G**CCCATTTG	2
**I**	CTGTTAATTTAAC**C**CCCATTTG	1

Alignment of pKLM/pLN spacer s3 with the corresponding proto-spacer region in 73 M5952 escape phage isolates grouped A - I. (Bold denotes single nucleotide mutation).

### CRISPR/Cas in Additional Lactococcal Strains

Lactococci from the DuPont collection were screened for the presence of the pKLM CRISPR/Cas locus using PCR primer sets derived from the repeat, leader, *cas1*, and 3′ trailer sequences (Table S1). Only 4 additional strains of over 400 examined tested positive for all reactions. Each of these 4 strains contains the full complement of pKLM *cas*/*csm* genes based on expanded PCR analysis. Sequence analysis showed that the CRISPR arrays were composed of the singular pKLM repeat. The CRISPR flanking regions that span *lch* through the leader sequence on one side and the trailer region into the truncated IS*1194* element on the other, are nearly identical. The *lch* gene in particular is 99% identical among the five characterized loci. In contrast, diversity was found in the number (from 4 to 15) and sequence content of spacers in each array. Twenty-three of the 26 new spacers are unique, three are shared, and one is duplicated within one array. Sequence analysis identified five spacers as having partial matches to lactococcal phage 949 or 936-type genomes.

### Adaptation

One feature of CRISPR/Cas is its ability to integrate new spacers in response to phage challenge [30]. Attempts to add spacers naturally to DGCC7192(pKLM) by challenge with escape phages derived from M5952 were repeatedly unsuccessful. A comparison of plasmid stability of pGK12 in 1403S versus 1403S(pLN) showed no tendency for plasmid loss in the CRISPR/Cas-containing strain, indicating no spacer against pGK12 was acquired.

### Engineering Spacer-directed Phage Resistance

In plasmid pG6E, loss of resistance to phages p2 (LM2345 host) and bIL170/P008 (1403S host) results from a deletion that includes spacer s4. We sought to determine if reinserting spacer s4 would restore the resistance phenotype. A plasmid containing a synthetic s4 was constructed (pRS4R) and integrated into the pG6 CRISPR locus. This created cointegrate plasmid, pG6::pRS4R, which, in addition to the pG6 array, contains a single spacer array composed of s4 in proper context with the *cas* genes (Figure 3). LM2345 containing pG6::pRS4R was resistant to phage p2. We then conjugally transferred pG6::pRS4R into 1403S. Transconjugants were resistant to phages bIL170 and P008. These results further correlate spacer-directed interference and demonstrate that CRISPR/Cas resistance can be programmed against specific phages.

## Discussion

This CRISPR/Cas system is, to our knowledge, the first described in *Lactococcus lactis*. Based on sequence and structural features, it is categorized as a Type III-A system [7], which is relatively rare in LABs [4], but found in microbes distantly related to lactococci, notably *E. italicus* and *S. epidermidis*. Our results characterizing the activity of this lactococcal CRISPR/Cas system against virulent phage complements studies of plasmid transfer inhibition and corroborates mechanistic studies of crRNA processing in staphylococci [21], [22], [33]. The lactococcal CRISPR repeat shows sequence conservation to other Type III-A CRISPRs, particularly at the 3′ end where the formation of a repeat stem-loop is essential for efficient interference activity in *S. epidermidis* [22]. The lactococcal spacers targeting phage apparently do not require 100% identity to confer phage resistance. It is not uncommon in CRISPR/Cas systems for targeting to be provided by a shorter “seed sequence” in the spacer [34], [35]. For the seventy-three M5952-escape phages examined, mutations circumventing s3-directed inhibition were localized within a 22-nt internal segment that is complementary to the start of *orf35* (in phage 4268), encoding a structural head protein [23].

As noted, the lactococcal *cas* gene cluster most closely resembles Type III loci with the exception that it does not contain a homologue to any known *cas2*. Instead, next to *cas1* is a gene with partial homology to the *relE*/*parE* toxin gene which we have designated *lch*. It is unclear if it could be functioning as a *cas2* despite lack of sequence conservation. It has been suggested that *cas2* evolved from a toxin gene, citing its homology to the VapDHi toxin protein [7]. This would be consistent with the speculation in archaea that association of CRISPR/Cas with toxin-antitoxin genes would stabilize the loci within the host genome [36]. It has also been reported that many organisms contain Type III CRISPRs lacking *cas1* and *cas2*, but all of these contain an additional CRISPR/Cas system in the genome, where *cas1* and *cas2* may function in *trans* [7]. *cas1* and *cas2* are required for spacer acquisition in *E. coli* [37]. Many spacer sequences among the few lactococcal CRISPRs analyzed have identity with lactococcal phages, suggesting the CRISPR is or has been adaptive in lactococci. The CRISPR diversity among the 5 strains analyzed, all containing the *lch* gene rather than a known *cas2*, may suggest the system is capable of spacer acquisition with its current Cas complement. Alternatively it may be explained as a series of spacer deletions by homologous recombination between direct repeats from a longer CRISPR array. It is possible that the system was active in an ancestor *Lactococcus*, such as those associated with plants in which plasmids are scarce [1], and that the acquisition ability was lost in the plasmid-rich *Lactococcus lactis* when it adapted to growth in milk. Due to the ancillary activity of CRISPR against plasmids, a non-adapting CRISPR/Cas system could be favorable for lactococci, allowing them to maintain plasmids encoding beneficial traits. Induction and regulation of the *L. lactis* CRISPR adaptation would increase its industrial utility as a phage resistance mechanism in starter strains.

CRISPR activity against plasmids may also explain its rarity in lactococci. Our previous screens for *S. thermophilus-*like type I and type II CRISPR systems in the DuPont *Lactococcus lactis* collection failed to detect any positives. A PCR-based screen of over 400 lactococci for the pKLM CRISPR/*cas* locus found its presence in only four additional strains confirming that the occurrence of CRISPR/Cas is strain specific [4]. This suggests that CRISPR/Cas may be more prevalent within bacterial genera or species than would be expected from an examination of available genomes.

The discovery of this lactococcal plasmid-encoded CRISPR/Cas is a function of the manner in which it was isolated; dependent on conjugative mobilization. Furthermore, the presence of numerous *IS*-elements on the plasmid suggests that CRISPR/Cas may have been acquired via transposition. It is undetermined if it is the norm or the exception in lactococci for the CRISPR/Cas to reside on a plasmid. It is a matter of speculation whether this is representative of a stable acquisition or a transition between loss or integration into the chromosome. Prior reports have established the presence of CRISPR/Cas on plasmids, and in some cases the plasmids also encode conjugation related proteins [47], [48]. Therefore, while CRISPR/Cas seems to be more commonly found on the chromosome based on examination of currently available genomic sequences, it is not unusual for a CRISPR/Cas to be plasmid-encoded.

Our data showing that this lactococcal CRISPR/Cas is plasmid-encoded and self-transmissible biologically confirms a route for CRISPR/Cas acquisition and dissemination. This corroborates bioinformatic evidence of horizontal transfer based on similar CRISPR/Cas loci present in non-phylogenetically related organisms and its residence on mobile elements [4], [47], [48]. Though a Type II CRISPR/Cas from *S. thermophilus* has been transferred to *E. coli* and shown to be functional, it was introduced by cloning the CRISPR/Cas into an *E. coli* plasmid [32]. We believe this to be the first biologic evidence of natural CRISPR/Cas mobility, which will enable further studies on dissemination and functionality in diverse microbes.

With respect to the hostile phage environment of industrial dairy processing, starter lactococci have evolved many different defensive systems. In this regard, the mobility of these phage resistance genes has been critical in the evolution and adaptation of lactococci in this application. Significantly, this CRISPR/Cas system, like many of the previously described lactococcal phage resistance mechanisms, is found on a self-transmissible plasmid, which we have exploited to transfer and characterize the system in additional lactococcal strains. The existing spacers target phages representative of common types in the industrial environment including 936- and 4268-like phage currently categorized with P335. Furthermore, the resistance could be engineered by direct introduction of a synthetic spacer against a specific phage. The initial demonstration of CRISPR/Cas phage interference in *S. thermophilus*, another food fermentation microbe, enabled a new avenue of protecting industrially important strains which has proven robust and highly adaptable. *L. lactis* has also been the focus of such natural engineering, however relying on varied mechanisms that, while initially efficacious, eventually are overcome by evolving phage. The discovery of a lactococcal CRISPR/Cas provides a potentially adaptable system that could be harnessed in response to evolving phage.

## Supporting Information

Table S1 **Primer sequences.** All primers were designed from pKLM sequence or phage M5952.
(DOCX)
