# Supplementary material for: Mobile CRISPR/Cas-Mediated Bacteriophage Resistance in Lactococcus lactis
Source: PLoS One. 2012 Dec 11;7(12):e51663. doi: 10.1371/journal.pone.0051663 (PMC3519859; doi:10.1371/journal.pone.0051663)
Supplement: Table S1 — Primer sequences. All primers were designed from pKLM sequence or phage M5952. (DOCX) [file pone.0051663.s001.docx]

**Supporting Information**

**Table S1.**

Primer sequences. All primers were designed from pKLM sequence or phage M5952.

| **Name** | **Sequence 5’ – 3’** | **Location** |
| --- | --- | --- |
| Cas genes 1F | TTAAGACTAGGATAGCACGAC | Upstream of cas10 (csm1) |
| Cas genes 1R | GTCAAGCACTCCTTCATTTGC | Middle of cas10 (csm1) |
| Cas genes 2F | GCAAATGAAGGAGTGCTTGAC | Middle of cas10 (csm1) |
| Cas genes 2R | GGACTACTGGGCTATCAACC | Beginning of csm3 |
| Cas genes 3F | GGTTGATAGCCCAGTAGTCC | Beginning of csm3 |
| Cas genes 3R | GTTGCATAAGTATCAGACTGG | End of csm4 |
| Cas genes 4F | CCAGTCTGATACTTATGCAAC | End of csm4 |
| Cas genes 4R | GATCTGTATCTCCTACGGCTG | Beginning of csm6 |
| Cas genes 4RB | GTAGCAGTGGTCCATCGTGA | Beginning of csm6 |
| Cas genes 5F | CAGCCGTAGGAGATACAGATC | Beginning of csm6 |
| Cas genes 5FB | TCACGATGGACCACTGCTAC | Beginning of csm6 |
| Cas genes 5R | CCTCATAGAAAGAGAACCAATGA | End of csm6 |
| Cas6F | TCATTGGTTCTCTTTCTATGAGG | End of csm6 |
| Cas6R | CTTTGAGCGTCATGTCACCT | End of cas6 |
| Cas1F | AGGTGACATGACGCTCAAAG | End of cas6 |
| Cas1R | CTCAAATACCAACACATCAACAA | End of cas1 |
| CR-F1 | TTGTTGATGTGTTGGTATTTGAG | End of cas1 |
| CR-R1 | CAATGGGCTAGATTGACCTA | Leader |
| CR-F2 | TAGGTCAATCTAGCCCATTG | Leader |
| CR-R2 | GAGTCTGTTGGTCTTATCCTTA | S9 |
| CR-F3 | TAAGGATAAGACCAACAGACTC | S9 |
| CR-R3 | GGTAGTTATTAGAGGTGCCC | Trailer |
| IS1194 | TACGTGTCCGTATTGAACATG | IS1194 |
| S4F | ATACGTTCTTTGAACCAAGCTTCAACTCCCTCGGAAAATACAACCGCTCCTCGATAAAAGGGGACGAGAACCATATGATTCAGGTATTGC | S4 + Repeat + Trailer |
| S4R | TCCGAGGGAGTTGAAGCTTGGTTCAAAGAACGTATGTTCTCGTCCCCTTTTATCGAGGAGCGGTTGTATTTAGAGAACTTTAAAAACGTG | S4 + Repeat + Leader |
| 4268 protospacer F | GTTGTTCTCTCAATGCGTCG | Bases 18468 - 18449 |
| 4268 protospacer R | TGGATGACAGCTCAAGATGC | Bases 18031 - 18050 |
